# Complement C3 Reduces Apoptosis via Interaction with the Intrinsic Apoptotic Pathway

**DOI:** 10.3390/cells12182282

**Published:** 2023-09-15

**Authors:** Zhou Fang, Haekyung Lee, Junying Liu, Karen A. Wong, Lewis M. Brown, Xiang Li, Alus M. Xiaoli, Fajun Yang, Ming Zhang

**Affiliations:** 1 Departments of Anesthesiology, SUNY Downstate Health Science University, 450 Clarkson Avenue, Brooklyn, NY 11203, USA; zhou.fang@downstate.edu (Z.F.); haekyung.lee@mountsinai.org (H.L.); junying.jolin@gmail.com (J.L.); karenawondmd@gmail.com (K.A.W.); xiang.li@downstate.edu (X.L.); 2 Quantitative Proteomics and Metabolomics Center, Department of Biological Sciences, Columbia University, New York, NY 10027, USA; lb2425@columbia.edu; 3 Department of Medicine/Endocrinology, Albert Einstein College of Medicine, Bronx, NY 10461, USA; alus.xiaoli@einsteinmed.edu (A.M.X.); fajun.yang@einsteinmed.org (F.Y.); 4 Departments of Cell Biology, SUNY Downstate Health Science University, 450 Clarkson Avenue, Brooklyn, NY 11203, USA

**Keywords:** ischemia/reperfusion injury (IRI), complement C3, apoptosis, cytochrome c, pro-caspase 3

## Abstract

Myocardial ischemia/reperfusion (I/R) elicits an acute inflammatory response involving complement factors. Recently, we reported that myocardial necrosis was decreased in complement C3^−/−^ mice after heart I/R. The current study used the same heart model to test the effect of C3 on myocardial apoptosis and investigated if C3 regulation of apoptosis occurred in human cardiomyocytes. Comparative proteomics analyses found that cytochrome c was present in the myocardial C3 complex of WT mice following I/R. Incubation of exogenous human C3 reduced apoptosis in a cell culture system of human cardiomyocytes that did not inherently express C3. In addition, human C3 inhibited the intrinsic apoptosis pathway in a cell-free apoptosis system. Finally, human pro-C3 was found to bind with an apoptotic factor, pro-caspase 3, in a cell-free system. Thus, we present firsthand evidence showing that C3 readily reduces myocardial apoptosis via interaction with the intrinsic apoptotic pathway.

## 1. Introduction

The complement system is a critical component of both the innate and adaptive immune systems that augments the function of antibodies and phagocytes [1]. Complement activation can be initiated through the classical, the alternative, and the lectin pathways [2,3,4,5,6,7,8,9,10]. Complement C3 is a central factor in the three activation pathways [2,3,4,5,6,7,8,9,10]. The three pathways converge at C3 and are followed by a common cascade [11]: C3 convertases yield C3a and additional C3b, with the latter forming C5a convertases that cleave C5 to C5a and C5b. C5b initiates membrane attack complex (MAC) formation (C5b-C9) [12,13].

C3 is mainly synthesized by hepatocytes [14], starting as pro-C3, which is a single polypeptide chain. Pro-C3 was proteolytically processed to two chains, α-chain (120 kDa) and β-chain (75 kDa) [15], which are linked by disulfate bonds and occur intracellularly [16]. After its processing, the double-chain C3 is secreted into circulation [17,18,19,20,21]. Spontaneous hydrolysis of C3, which takes place in plasma [22], generates its hydrolytic product C3(H_2_O). Further enzymatic cleavage of the C3 alpha-chain, which may occur intracellularly and extracellularly [23], generates fragments of the overall molecule, termed C3a, C3b, C3c, C3d,g, C3d, and C3g [10].

Myocardial ischemia/reperfusion (I/R) elicits an acute inflammatory response involving complement factors of the innate immune system [24,25,26]. Complement inhibitors, which have the potential to limit inflammation, showed promise in preclinical studies [27,28,29,30,31]. However, limited positive results were obtained with the few inhibitors studied in clinical trials of myocardial I/R injury [26]. In particular, anti-complement C5 (Pexelizumab) failed to meet the primary endpoints in cardiac patients who had undergone coronary artery bypass graft surgery [32] or percutaneous transluminal coronary intervention [33]. One explanation of the negative clinical trial results is that they targeted downstream components in the complement pathway, e.g., C5, leaving earlier activators such as C3 unaffected. Activation of the earlier complement factors would have affected pathways such as those leading to cell death with the potential to influence the outcome.

Previous animal studies by us and others showed that under pathological conditions such as I/R injury, circulation C3 was deposited in the ischemic myocardium flooded with oxygenated blood upon reperfusion [34,35]. We recently reported that in the mouse heart model, myocardial necrosis was decreased in C3^−/−^ mice [36]. This implied that in WT mice during I/R, C3 acts to promote necrosis. Besides necrosis, basic research has indicated that apoptosis plays an important role in cardiac I/R injury [37,38,39]. Whether C3 is involved in apoptosis during myocardial I/R is unclear. The current study used the same heart model to test the effect of C3 on myocardial apoptosis and investigated if C3 regulation of apoptosis occurred in human cardiomyocytes.

## 2. Materials and Methods

### 2.1. Mouse Model of Myocardial I/R Injury

Complement C3 knockout (C3^−/−^) mice and WT (C57BL/6) mouse strains were obtained from Jackson Laboratory (Bar Harbor, ME, USA) and maintained at SUNY Downstate Medical Center Department of Laboratory Animal Resources. Genotyping was provided by GeneTyper (New York, NY, USA). Male mice were used at 10–12 weeks of age (weight 26–30 g) in accordance with the requirements of the NIH and the Institutional Animal Care and Use Committee (IACUC) of SUNY Downstate Medical Center. The protocol was approved by the IACUC of SUNY Downstate Medical Center (Approval #11-10276).

We employed an established myocardial I/R injury model [35,40]. Mice were anesthetized using sodium pentobarbital (60 mg/kg, i.p.), intubated, and ventilated with a mouse ventilator (Harvard Apparatus, MA, USA). Following sternotomy, the left anterior descending artery (LAD) was ligated for 1 h; occlusion of the LAD was confirmed by the appropriate color change of myocardial tissue and the ST elevation on ECG; reperfusion was verified by the reversed color change of the left ventricle and the appropriate ECG changes. Postoperative management included fluid replacement with normal saline and pain relief with the analgesic, buprenorphine (0.1 mg/kg, intramuscularly). The mice were sacrificed, and the hearts were harvested at 3 h of reperfusion for proteomic study and 24 h of reperfusion for histopathologic analyses.

### 2.2. Comparative Proteomics

Comparative proteomics with label-free shotgun profiling was carried out at Quantitative Proteomics Center, Columbia University. Proteins in heart samples were solubilized using Rapigest (Waters Corp., MA, USA) and digested with trypsin. Peptides were separated in a 120 min LC run on a 0.75 µm ID × 25 cm reverse phase 1.7 µm particle diameter C18 column and analyzed on a Synapt Q-TOF mass spectrometer. MS and MSE spectra were analyzed using an MSE/IdentityE algorithm. In addition, the Elucidator Protein Expression Data Analysis System software version 3.2 (Rosetta Biosoftware, MA, USA), which can import IdentityE data, was used for advanced multivariate statistical analysis, false discovery control, principal components analysis, multidimensional scaling, and cluster analysis. To identify the C3-specific signaling network in necrosis, proteins identified from proteomics were mapped to existing biological networks by Ingenuity Pathway Analysis (QIAGEN, CA, USA)

### 2.3. ELISA Measurement of Cytochrome c in the C3-Immunocomplex

A sandwich ELISA was used to verify the presence of cytochrome c in the C3 complex. The wells of a 96-well plate were coated with an anti-C3 Ab (Complement Technology, TX, USA) to capture the C3 complex in the heart cytosolic fractions. After washing to remove unbound factors and blocking with BSA, an anti-cytochrome c Ab (Enzo, NY, USA) was used to detect the presence of cytochrome c.

### 2.4. Western Blotting of Cytochrome c

Cytosolic fractions were isolated using a published method [41]. Briefly, hearts were homogenized in a Dounce homogenizer in cytosolic extraction buffer. Lysates were centrifuged (600× *g*, 10 min, 4 °C), and the cytosolic extract (supernatant) was collected and further centrifuged (10,000× *g*, 10 min, 4 °C). The protein (20 μg) from each cytosolic extract was analyzed by Western blotting following 12% SDS-PAGE. Membranes were probed with a sheep anti-mouse cytochrome c Ab (Enzo, NY, USA) followed by a donkey anti-sheep IgG coupled to horse radish peroxidase (R&D, MN, USA) and developed with an ECL Western blotting kit (Thermo Scientific, NJ, USA). The intensities of the cytochrome c bands were normalized to that of constitutively expressed β-actin and expressed as relative intensity. Quantification of bands of interest was carried out using the ImageJ program (NIH, Bethesda, MD, USA).

### 2.5. Cell Lines, Abs, Molecular Cloning Construct, and Proteins

The AC16 human cardiomyocyte cell line (Millipore, MA, USA) was grown in DMEM:F12 with 10% heat-inactivated FBS, 1% penicillin, and 1% L-Glutamine. The 293 T cell line was grown in DMEM with 10% heat-inactivated FBS and 1% penicillin. Both cell lines were maintained at 37 °C in 5% CO_2_. Rabbit anti-human C3c (F0201) polyclonal Ab was obtained from Agilent Dako (Santa Clara, CA, USA). Goat anti-human C3 (A213) polyclonal Ab was obtained from Complement Technologies (Tyler, TX, USA). Rabbit anti-cytochrome c polyclonal Ab was obtained from Cell Signaling (Danvers, MA, USA). Rabbit anti-caspase 3 polyclonal Ab was obtained from Santa Cruz Biotechnology (Santa Cruz, CA, USA). The full length human complement C3 expression ORF clone, whose C3 gene cDNA ORF clone sequences were retrieved from the NCBI Reference Sequence Database with the vector pcDNA 3.1+/C-(K)DYK, was obtained from GenScript (Piscataway, NJ, USA) (Supplementary Figure S4).

### 2.6. qPCR for C3’s mRNA Expression

C3 mRNA expression were studied by qPCR, and a positive control that internally expressed C3 was included by using Huh 7 human liver cell line [42].

### 2.7. H_2_O_2_-Induced Apoptosis in Human Cardiomyocytes

Hydrogen-peroxide-induced (H_2_O_2_, Sigma-Aldrich, MO, USA) apoptosis was performed in confluent AC16 cells. Cells were incubated with H_2_O_2_ for 60 min and then washed with PBS. Cells were then incubated in the presence or absence of purified human C3 for 3 h. Apoptotic cell death was detected by Dr. Kulkarni at Washington University using FACS analyses of Annexin-V and propidium iodide staining, as reported previously [43].

### 2.8. Cell-Free Apoptosis Analyses

A cell-free apoptosis system was employed [44,45]. Cytosolic fractions from xenopus oocytes extracts were prepared and graciously supplied by Dr. Leta Nutt at St. Jude Hospital [44,45]. Purified C3 was pre-incubated with either purified cytochrome c (Sigma, MO, USA) or a purified cytosolic fraction for 1 h, followed by either addition of purified cytosol from xenopus or purified cytochrome c, and incubation for one hour. Apoptosis activities were detected using substrate from the Caspase-3 Glo Apoptosis kit (Promega, WI, USA).

### 2.9. Pull-Down Assay to Detect Apoptotic Factor(s) Interacted with C3

#### 2.9.1. Transfection and Overexpression of Human C3 in 293 T Cell Line

The 293 T cells were split and placed in a 10 cm dish 4 h before transfection. The DNA/CaPO4 mix for transfection was prepared as follows: 10 μg of total DNA, 62 μL of 2M CaCl_2_, with additional ddH2O up to 500 μL total volume. This mixture was added to 500 μL of 2X HBS at RT. DNA/CaCl_2_/HBS mixture was sat at room temperature for 25 min. Then, the mixture was mixed again, followed by sprinkling the entire mixture over the plate of cells. The cells were cultured in the medium with the transfection mixture for 36 h to 48 h followed by collection of the cells.

#### 2.9.2. Apoptotic Cell Lysate Preparation

To prepare apoptotic cell lysate of human cardiomyocytes, AC16 cells were incubated with 500 μM H_2_O_2_ (Sigma-Aldrich (St. Louis, MO, USA)) for 30 min and then washed with PBS. The cells were collected. Cell lysate was prepared by resuspending cell pellets in IP buffer (0.2% NP-40, 10% Glycerol, 10 mM Tris-HCl at pH 8.0, and 100 mM KCl) with 1% HaltTM Protease Inhibitor Cocktail (100×) (78430, Thermo Scientific, Waltham, MA, USA) and incubating for 10 min on ice. The resulting lysates were then centrifuged for 10 min at 8000× *g* and supernatants were collected, aliquoted, and stored at −80 °C. The same procedure for cell lysate was applied for the 273 T cells.

#### 2.9.3. Identification of Apoptotic Factor(s) Binding to C3

This pull-down method is based on the binding affinity between antigen and antibody and the subsequent isolation of the capture/target complex by precipitation [46,47,48]. The anti-Flag antibody precoated beads were incubated with transfected 293 T cell lysate for 3 h at 4 °C. After incubation, the beads were collected and washed once with the IP buffer. After washing, the beads were incubated with AC16 cell lysate, which was treated with H_2_O_2_ for 3 h at 4 °C. The beads were washed 4 times after incubation, followed by elution of the protein from the beads. The proteins in the protein complex were identified by Western blot.

### 2.10. Statistical Analysis

Statistical analyses were performed using IBM SPSS Software version 20 (IBM Corp., NY, USA). An independent t-test with two tails and unequal variances was used to determine the statistical significance of differences between the results of experimental and control groups. Descriptive data were summarized as mean ± standard error of mean.

## 3. Results

### 3.1. Identification of Cytochrome c in a Myocardial C3 Complex Following I/R Using Comparative Proteomics

**Figure 1:**
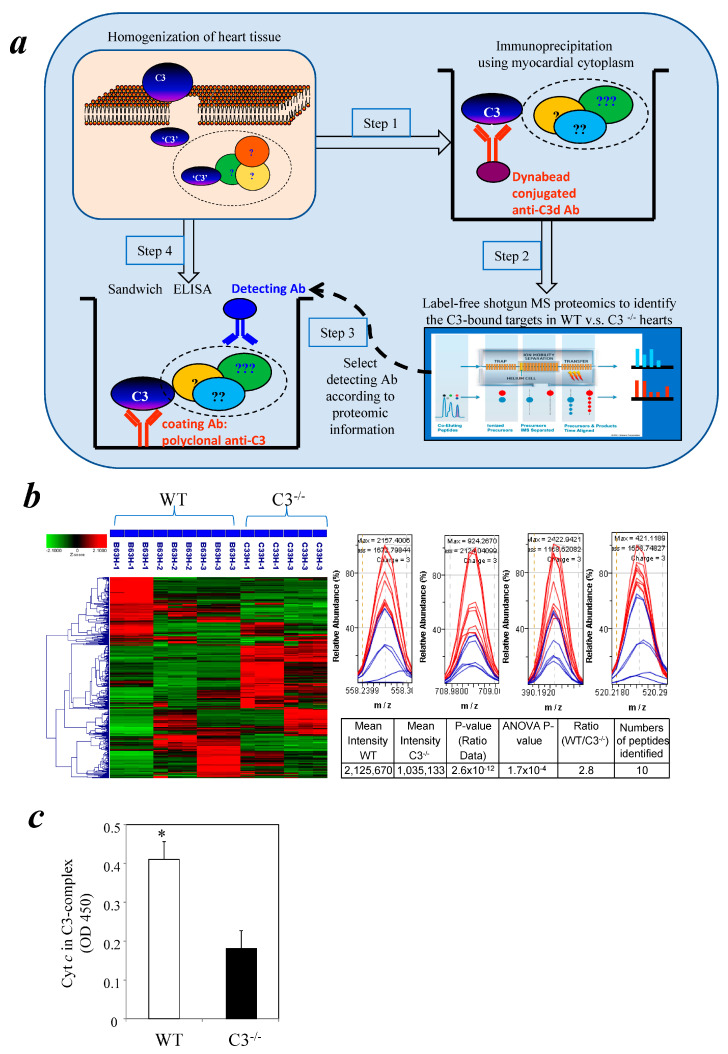
Comparative proteomics identified cytochrome c in C3-containing complexes formed during myocardial I/R. (**a**) Schematic diagram depicts the steps of experimental approaches. Step 1: hearts were harvested and homogenized from WT and C3^−/−^ mice, which were subjected to 1 h ischemia/3 h reperfusion; C3-containing complexes in heart lysates were immunoprecipitated with an anti-C3d Ab. Step 2: the C3-immunoprecipitated complexes were digested with trypsin and analyzed by label-free shotgun LC–mass spectrometry. Step 3: select detecting Ab according to proteomic information. Step 4: sandwich ELISA to confirm the specific protein in C3 complex. (**b**) Left panel: Chromatograms were recorded for each biological replicate in Resolution/Ion Mobility mode. Agglomerative hierarchical clusters of Z-score transformed intensity data were processed by the Elucidator program for all LC–MS chromatograms. Z-score coloration indicates protein abundance in WT compared with C3^−/−^ samples (red, higher abundance; green, lower abundance; black, equal abundance). Right panel histograms: Comparison of cytochrome c peptide signals of specific m/z ratios between WT and C3^−/−^ mice. Red and blue lines represent peptide signals from WT and C3^−/−^ mice, respectively. At bottom-right is the summary table of the cytochrome c peptide signal comparisons of WT and C3^−/−^ mice. (**c**) Cytochrome c is present in a cytosolic C3-binding complex following 1 h ischemia and 3 h reperfusion. Myocardial cytosolic factions were isolated. C3-binding complexes in the myocardial cytosolic fractions were captured with a polyclonal anti-C3 Ab bound to the surface of microplates. An anti-cytochrome c Ab was used to detect cytochrome c in C3-binding complexes (*n* = 3 mice/group). * *p* < 0.05.

The current study used an established mouse heart I/R model [36] to investigate the effect of C3 on myocardial apoptosis. A comparative proteomic approach was used in our investigation. Our earlier study showed that in the mouse myocardial I/R model, C3 deposition becomes significant at 3 h reperfusion [35]. Similar C3 deposition was also reported by others in a rat model [49]. Thus, heart lysates were prepared from WT and C3^−/−^ mice after 1 h ischemia/3 h reperfusion to capture the factor(s) interacting with C3 at the early phase. C3-containing protein complexes were isolated using anti-C3d conjugated Dynabeads (Life Technologies, CA, USA). The complexes were analyzed by comparative proteomics (Figure 1a). A total of 57 proteins were identified as being preferentially present in complexes with C3 in WT mice compared with C3^−/−^ mice (WT:C3^−/−^ ratio score >2, *p* < 0.05) (Figure 1b). Of these 57 proteins, only one protein, cytochrome c, is known to be directly involved in cell death (Supplementary Table S1). Pathway analysis predicted that cytochrome c is in a protein network that includes C3 (Supplementary Figure S1).

### 3.2. Detection of Cytochrome c in a Cytosolic C3 Complex Following I/R by Immunoassay

To confirm the comparative proteomic results, we developed a sandwich ELISA to determine if cytochrome c and C3 interacted in the cytosol of post-I/R cardiomyocytes. Cytosolic fractions were isolated from the hearts of WT and C3^−/−^ mice after 3 h reperfusion and added to a microplate coated with a polyclonal anti-native C3 Ab. Cytochrome c was detected by an Ab against cytochrome c. There was significant binding of cytochrome c to C3 in WT mice compared with the background levels in C3^−/−^ mice (Figure 1c).

### 3.3. Similar Levels of Cytochrome c in Myocardial Cytosol of C3^−/−^ and WT Mice after I/R

It is well known that anti-apoptotic signaling molecules such as Bcl-2 and Bcl-xL prevent cytochrome c release from mitochondria and, thus, inhibit apoptosis [50]. Conversely, pro-apoptotic molecules, e.g., BAX and BH3-only proteins [51], enhance cytochrome c release and promote apoptosis. Thus, the question is raised as to whether C3^−/−^ mice have increased cytochrome c released into the cytosol after I/R.

To investigate this possibility, we isolated myocardial cytosolic fractions from C3^−/−^ and WT mice following I/R and compared the cytochrome c levels by Western blotting. There were no significant differences in the cytochrome c levels in C3^−/−^ and WT mice at each time point (Supplementary Figure S2). Thus, the mitochondrial response to I/R stress, in terms of cytochrome c released, is similar in C3^−/−^ mice and WT mice.

### 3.4. Reduction of Apoptosis in AC16 Cardiomyocytes by Exogenous C3

**Figure 2:**
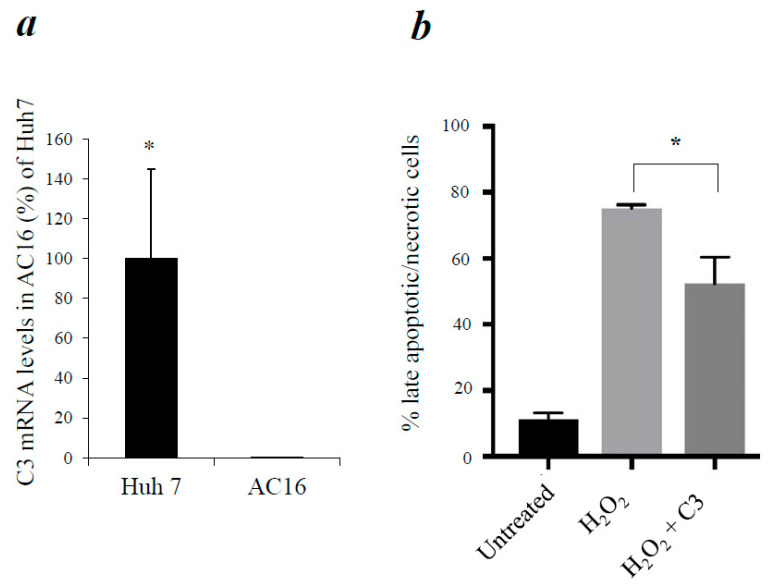
Exposure to exogenous C3 reduced apoptosis in cardiomyocytes. (**a**) C3 mRNA levels in AC16 cardiomyocytes were measured by qPCR. Huh 7 human liver cell line was used as a positive control of C3 expression. Error bars indicate SEM; * *p* < 0.05. (**b**) AC16 cells were incubated with H_2_O_2_ for 60 min and then washed with PBS. Cells were then incubated in the presence or absence of purified C3 for 3 h. Apoptotic cell death was detected by FACS analyses of Annexin V-Propidium Iodide staining.

To study if C3 has the same anti-apoptotic effect in human cardiomyocytes, we first analyzed if AC16 cells express C3 internally. C3 mRNA expressions were determined by qPCR, and a positive control that internally expressed C3 was included (a human liver cell line, Huh 7) [42]. High levels of C3 expression were detected in Huh 7 cells, but AC16 cells did not have detectable C3 mRNA (Figure 2a). Then, we induced apoptosis in AC16 cells by H_2_O_2_ treatment, as reported in [52]. When AC16 cells were incubated with a high dose of H_2_O_2_ for 60 min, they underwent apoptosis as expected (Figure 2b). However, addition of C3 into the cell culture significantly decreased apoptosis of AC16 cells (Figure 2b). Therefore, although AC16 cells do not express C3, exposure of the exogenous C3 could reduce apoptosis in these cells under oxidative stress conditions.

### 3.5. Inhibition of the Intrinsic Apoptosis Pathway by C3 in a Cell-Free System

Our study found that cytochrome c was present in the C3-containing protein complexes of hearts in a murine I/R injury model, suggesting that C3 may influence apoptosis by interacting with cytochrome c in the intrinsic apoptotic pathway (Figure 1). Basic research has suggested that in the intrinsic apoptosis pathway, cytochrome c released from mitochondria binds to Apaf-1 to form the apoptosome by recruiting caspase-9 [53,54], which further activates downstream caspases-3 and -7, leading to apoptosis [55].

To investigate the mechanism underlying C3’s interaction with cytochrome c in the intrinsic apoptosis pathway, we employed a cell-free apoptosis system [44,45]. As outlined in Supplementary Figure S3, we carried out experimental approaches as follows: (1) pre-incubation of human C3 with cytochrome c followed by addition of the cytosolic fraction without mitochondria (containing factors of the intrinsic apoptosis pathway except endogenous cytochrome c); (2) pre-incubation of C3 with purified cytosol without mitochondria followed by addition of cytochrome c. Our hypotheses are as follows: (i) if C3 binds cytochrome c directly, Approach #1 will show reduction of apoptosis when C3 is pre-incubated with cytochrome c while Approach #2 will not show apoptosis reduction; (ii) on the other hand, if C3 binds to factor(s) downstream of cytochrome c in the intrinsic apoptosis pathway, Approach #1 will not block apoptosis while Approach #2 will block apoptosis when C3 is pre-incubated with purified cytosol followed by the addition of cytochrome c.

**Figure 3:**
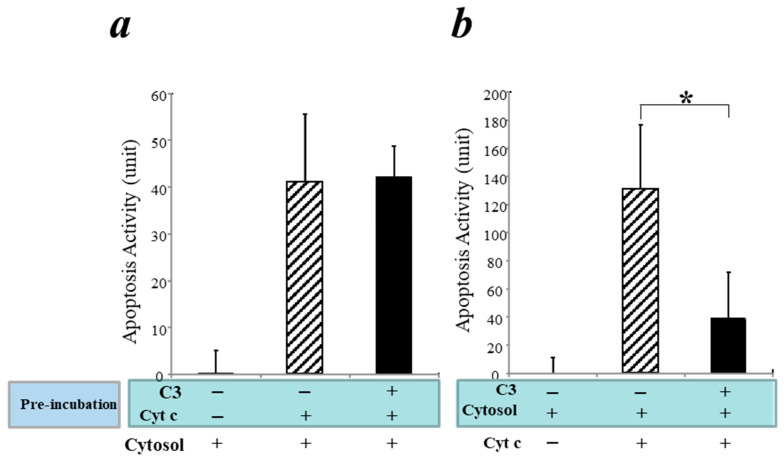
In a cell-free apoptosis system, C3 interacts with factor(s) downstream of cytochrome c and blocks cytochrome c-mediated apoptosis. (**a**) Purified C3 was pre-incubated with purified cytochrome c for one hour, followed by addition of purified cytosol from xenopus and incubation for one hour. Apoptosis activities were detected using substrate from the Caspase-3 Glo Apoptosis kit. (**b**) Purified C3 was pre-incubated with purified cytosol for one hour, followed by addition of cytochrome c and incubation for one hour. Apoptosis activities were detected as in (**a**). * *p* < 0.05.

In a control experiment without C3, incubation of cytochrome c with purified cytosol induced apoptosis as expected, confirming that this cell-free system worked as reported [44,45] (Figure 3a,b, hatched bars). As a negative control, purified cytosol alone could not induce apoptosis without cytochrome c (Figure 3a,b, left-side bars). Pre-incubation of C3 with cytochrome c did not block cytochrome c-mediated apoptosis (Figure 3a, black bar). However, pre-incubation of C3 with purified cytosol blocked the cytochrome c-mediated apoptosis (Figure 3b, black bar). Thus, these data indicate that C3 does not bind cytochrome c directly but, rather, interacts with downstream apoptotic factor(s) in the cytosol in the intrinsic apoptosis pathway.

### 3.6. Pro-C3 Binding with Pro-Caspase 3 in a Cell-Free System

Previous animal studies by us and others have shown that under pathological conditions such as I/R injury, circulation C3 was deposited in the ischemic myocardium flooded with oxygenated blood upon reperfusion [34,35], which could be a reason to reduce myocardial apoptosis. To further study the role of C3 in human cardiomyocyte apoptosis, we investigated if C3 is able to interact with the intracellular proteins related to apoptosis. We employed a method of antibody pull-down to identify potential intracellular targets bound to C3. To obtain a large amount of tagged-human C3 for the pull-down assay, we transfected 293 T cells with a plasmid construct that overexpressed a full-length of single-chain pro-C3 with a Flag-tag at its C-terminal (Flag-pro-C3; Supplementary Figure S4). The overexpressed Flag-pro-C3 was captured by beads pre-coated with anti-Flag antibody and served as the “bait” in the pull-down assay. The “prey” would be apoptosis-related protein(s) in the cell lysate of AC16 cells treated with H_2_O_2_, which induced apoptosis in these cells [52].

**Figure 4:**
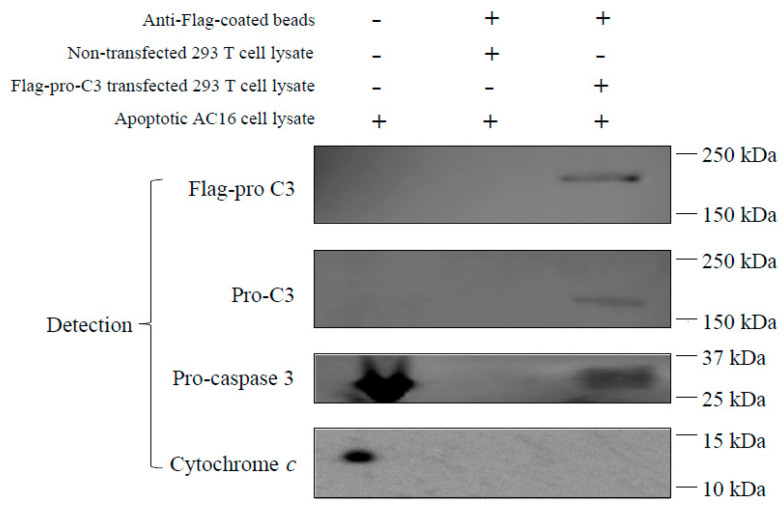
Pro-C3 binding with pro-caspase 3 in a cell-free system. Lane 1: AC16 cells were treated with 500 mM H_2_O_2_ for 30 min. Cell lysate was prepared as described in Methods section. Lane 2: Anti-flag antibody coated beads were incubated with cell lysate of non-transfected 293 T cells (without Flag-pro-C3) and then with AC16 cell lysate. The pull-down materials were used for analyses. Lane 3: Anti-Flag antibody coated beads were incubated with cell lysate of transfected 293 T cells (containing Flag-pro-C3) and then with cell lysate of AC16 cells. The pull-down materials were then used for analyses.

Pro-C3 proteins were readily detected in the pull-down complex using anti-Flag or anti-pro-C3 antibodies (Figure 4, Lane 3), demonstrating that transfection, overexpression, and pull-down of Flag-pro-C3 proteins were successful. As a negative control, IP buffer was used instead of transfected 293 T cell lysate to incubate with the anti-Flag beads (Figure 4, Lane 2). No C3 proteins were detected in the H_2_O_2_-treated AC16 cell lysate (Figure 4, Lane 1), confirming that AC16 cells do not produce C3 inherently. When Flag-pro-C3 bound beads were incubated with the AC16 cell lysate to pull down proteins interacting with pro-C3, we could detect pro-caspase 3 in the pro-C3-binding complex (Figure 4, Lane 3). Although both pro-caspase 3 and cytochrome c are abundant in H_2_O_2_-treated AC16 cell lysates (Figure 4, Lane 1), pro-C3 did not pull down any cytochrome c proteins (Figure 4, Lane 3), suggesting that cytochrome c is not present in the pro-C3-binding complex.

## 4. Discussion

Our results indicated that C3 interacts and blocks the intrinsic apoptotic pathway in cardiomyocytes during oxidative stress. Taken together with our earlier report [36], these findings imply that in WT mice during myocardial I/R, C3 acts to promote necrosis and block apoptosis in the heart.

It is of note that such a dual role of C3 in regulating cell death seems to be contrary to the current dogma regarding cardiac necrosis and apoptosis—both have a negative impact on the heart. Nevertheless, this dual role of C3 does not preclude the possibility that other factors can block (or promote) both apoptosis and necrosis simultaneously. Regarding the long-term impact, we have shown that 4 weeks after the initial IR, C3^−/−^ mice had better LVEDD than WT mice [36], although there was no statistical difference in EF or FS between C3^−/−^ and WT mice (Zhang, unpublished data). In addition, C3^−/−^ mice had less cardiac fibrosis than WT mice 4 weeks after initial I/R [36]. Thus, our data suggest that, overall, C3 causes cardiac fibrosis in the long term. As in any research, new questions often arise after the initial finding, and we acknowledge that our current study opens a question regarding the contributions from C3-promoted necrosis vs. C3-regulated apoptosis to long-term cardiac remodeling after IR. Further research may help solve this question.

The results from proteomics and the cytosol ELISA assay indicate that C3 can interact directly with cytochrome c in the cytosol of cardiomyocytes during myocardial I/R (Figure 1). As far as we are aware, there are no published reports for a direct interaction between the two. This experimental finding is the first evidence for a C3 complex containing cytochrome c.

It is relevant to note that cytochrome c is normally anchored to the inner mitochondrial membrane but is released to the cytosol by various pro-apoptotic signals and induces apoptosis by activation of caspases [55,56]. The identification of cytochrome c in a C3-containing complex suggested that cytosol-located, activated C3 might interfere with the function of cytochrome c in apoptosis.

Our results suggest that the post-I/R C3^−/−^ hearts did not have an increase in the amount of cytochrome c released into the cytosol. It is possible that binding of C3 to cytosolic cytochrome c in WT mice sequesters cytochrome c, thus reducing the number of cells that complete apoptosis. The result from our recent report indicates that necrosis is favored in WT mice expressing C3 [36]. Therefore, during I/R, based on our results, C3 minimizes apoptosis and promotes necrosis. These results raise the possibility of a new mechanism affecting cell death relevant to pathologic conditions such as ischemia: a circulating innate immune factor enters cardiomyocytes, interacts directly with intracellular factor(s), and influences the types of cell death that occur.

Our results showed that although human cardiomyocytes (AC16 cells) did not express C3, incubation of exogenous C3 reduced apoptosis in a cell culture system of cardiomyocytes (Figure 2) as well as in a cell-free apoptosis system (Figure 3). Furthermore, pro-C3 was found to bind with an apoptotic factor, pro-caspase 3, in a cell-free system (Figure 4). Thus, we presented firsthand evidence that exogenous C3 may interact with factor(s) in the intrinsic apoptotic pathway to inhibit apoptosis in cardiomyocytes.

Regarding how the exogenous C3 can interact with apoptotic factor(s) in cytosol, our pilot studies suggested that although AC16 cells did not produce matured C3, they readily took up the exogenous native C3 from extracellular milieu (Zhou, unpublished results). C3 uptake has been reported in human immune cells [57], airway epithelial cells [43], and retinal epithelial cells [58]. In particular, C3 uptake protects human airway epithelial cells from H_2_O_2_-induced cell death [43]. Therefore, the anti-apoptotic function of C3 could be carried out by the exogenous C3 after its uptake by AC16 cells.

Our results showed that cytochrome c is present in the C3-binding complex in a murine myocardial I/R model, in which apoptosis is regulated. However, our current results suggest that human C3 does not directly interact with cytochrome c but, rather, downstream factor(s) in the intrinsic apoptosis pathway (Figure 3). One explanation is that cytochrome c may be present in the apoptotic complex in human cardiomyocytes to initiate the intrinsic apoptosis pathway but the continuation of apoptosis process is blocked when C3 binds to a downstream factor in the pathway. An alternative explanation is that species differences may be manifested in the interactions between C3 and apoptotic factor(s) in mice versus humans.

We attempted to use the pull-down assay to identify the potential apoptotic factor(s) that C3 directly interacts with. Our results showed that pro-C3 can bind with pro-caspase 3 (Figure 4). While this finding is novel and interesting, it also opens some new scientific queries. For instance, pro-C3 is typically known as a single-chain protein inside the cell before being enzymatically processed into two chains linked by disulfate bonds. Traditionally, pro-C3 is thought to be not functionally active; however, our finding of pro-C3 binding with pro-caspase 3 suggests that it may function inside the cell by regulating apoptosis. Nevertheless, the purified C3 we used in anti-apoptotic experiments (Figure 2, Figure 3 and Figure 4) were from human plasma and, thus, in the double-chain form. It remains to be determined if the double-chain form of C3 binds to specific factor(s) in the intrinsic apoptosis pathway similar to that of the single-chain form. Future research on this line may provide more insights.

Accumulative evidence indicates that the role of C3 in regulating apoptosis appears to be pertinent to specific cell types and pathological conditions. For instance, C3 exhibited pro-apoptotic effects in murine retina during photo-oxidative damage [59] and in primate kidney during hemorrhagic shock [60]. On the other hand, C3 was also reported to have an anti-apoptotic function. For example, C3 can protect pancreatic β-cells from cytokine-induced apoptosis [61] and possibly interact with the autophagy-associated protein ATG16L1 [62,63]. In addition, C3 seemed to protect airway epithelial cells in a cigarette smoking model [64] and airway epithelial cells readily took up C3 from exogenous sources to mitigate apoptosis [43]. C3 was also reported to inhibit apoptosis in bullous-like skin inflammation [65]. Taken together with our current findings, C3 may act as a double-edged sword in regulating cell apoptosis depending on the pathologic conditions.

Whether C3-regulated apoptosis interacts with other established mechanisms of apoptosis is still unknown. It is possible that at a certain point of apoptotic signal transduction in cardiomyocytes, the C3-mediated mechanism may overlap with other mechanisms. For instance, Fas-mediated signal transduction can cause the release of cytochrome c and activation of caspase cascade [66], which may be a cross-point with the C3-mediated mechanism.

I/R injury has been associated with non-flow phenomenon (NFP) in coronary artery disease, which predicts a poor outcome despite “apparent” angiographic optimal percutaneous coronary intervention (PCI) [67]. Platelet-related microembolization and microvascular inflammation in the affected artery play a pivotal role in NFP [67]. It will be interesting for future research to explore if C3 contributes to microembolization and microvascular inflammation in NFP.

## Data Availability

The data presented in this study are available in this current article.

## Supplementary Materials

The following supporting information can be downloaded at: https://www.mdpi.com/article/10.3390/cells12182282/s1, Figure S1: Pathway analysis of protein networks associated with C3; Figure S2: Cytochrome c levels in C3^−/−^ and WT myocardial cytosols after I/R; Figure S3: Schematic diagram depicting the hypothesis and model of the possibilities/results for C3 interaction with factor(s) in the intrinsic apoptotic pathway; Figure S4: pcDNA3.1+/C-(K)DYK vector map; Table S1: Comparative Proteomics Analyses of proteins in C3-binding complex.
