# Supplementary material for: Complement C3 Reduces Apoptosis via Interaction with the Intrinsic Apoptotic Pathway
Source: Cells. 2023 Sep 15;12(18):2282. doi: 10.3390/cells12182282 (PMC10528058; doi:10.3390/cells12182282)
Supplement: Supplementary file 1 [file cells-12-02282-s001.zip › cells-2570218-supplementary.pdf]

Supplementary Table S1. Comparative Proteomics Analyses of proteins in C3-binding complex

Primary Protein Name	Protein Description	P-value (Ratio Data)	ANOVA P-value	Ratio (WT/KO)	# Peptides
B2RQQ1_MOUSE	MCG133649, isoform CRA_a GN=Myh6; cardiac muscle, alpha; alpha-MHC	0.01	0.03	2.2	125
CO3_MOUSE	Complement C3, Alternative initiation; Cleavage on pair of basic residues; Complement alternate pathway; Complement pathway; Direct protein sequencing; Disulfide bond; Glycoprotein; Immunity; Inflammatory response; Innate immunity; Phosphoprotein; Secreted; Signal; Thioester bond	1.8E-07	2.8E-03	2.3	47
Q497E4_MOUSE	Actin alpha cardiac GN=Actc1	2.4E-20	1.9E-04	2.2	36
MYL3_MOUSE	Myosin light chain 3 GN=Myl3	1.2E-09	4.3E-04	2.7	32
ALBU_MOUSE	Serum albumin GN=Alb	4.2E-07	6.9E-03	2.4	24
Q545Y3_MOUSE	Tropomyosin 1, alpha, isoform CRA_1 GN=Tpm1	6.2E-03	5.6E-03	18.5	22
ACTN2_MOUSE	Alpha-actinin-2 GN=Actn2	3.9E-03	0.05	2.1	21
B1AR69_MOUSE	Myosin, heavy polypeptide 13, skeletal muscle GN=Myh13	2.0E-06	0.04	2.7	19
MYH3_MOUSE	Myosin-3 GN=Myh3	4.3E-03	0.08	2.3	19
MYH7B_MOUSE	Myosin-7B GN=Myh7b	4.4E-10	6.4E-04	2.6	13
LDB3_MOUSE	Isoform Oracle 2 of LIM domain-binding protein 3 GN=Ldb3	5.2E-32	0.02	2.0	11
CYC_MOUSE	Cytochrome c, somatic GN=Cycs	2.6E-12	1.7E-04	2.8	10
Q5SX41_MOUSE	Myosin, heavy polypeptide 2, skeletal muscle, adult GN=Myh2	2.1E-03	5.2E-04	5.8	8
E9PZF0_MOUSE	Nucleoside diphosphate kinase GN=Nme2	0	0.30	2.6	8
Q3UVB1_MOUSE	Myoglobin, isoform CRA_a GN=Mb	1.2E-06	8.5E-03	2.0	7
NDUS6_MOUSE	NADH dehydrogenase [ubiquinone] iron-sulfur protein 6, mitochondrial GN=Ndufs6	3.9E-03	0.18	2.0	7
ECH1_MOUSE	Delta(3,5)-Delta(2,4)-dienoyl-CoA isomerase, mitochondrial GN=Ech1	9.8E-22	4.7E-03	3.2	6
NDUAA_MOUSE	NADH dehydrogenase [ubiquinone] 1 alpha subcomplex subunit 10, mitochondrial GN=Ndufa10	5.3E-31	0.04	4.1	5
B1ATS4_MOUSE	ATPase, Ca++ transporting, ubiquitous GN=Atp2a3	1.3E-35	1.4E-06	3.2	5
K2C75_MOUSE	Keratin, type II cytoskeletal 75 GN=Krt75	4.2E-33	8.0E-07	8.7	4
B1ASG5_MOUSE	Ubiquinol-cytochrome c reductase hinge protein GN=Uqcrh	1.5E-10	7.1E-07	7.7	4
Q497F1_MOUSE	Troponin I, cardiac 3 GN=Tnni3	9.8E-05	5.9E-04	4.4	4
E9PYX4_MOUSE	Glyceraldehyde-3-phosphate dehydrogenase	4.1E-08	2.8E-03	3.6	4
K2C1_MOUSE	Keratin, type II cytoskeletal 1 GN=Krt1	5.3E-08	3.6E-04	3.3	4
K22O_MOUSE	Keratin, type II cytoskeletal 2 oral GN=Krt76	2.1E-03	0.07	3.0	4
TBA4A_MOUSE	Tubulin alpha-4A chain GN=Tuba4a	1.9E-11	3.8E-03	2.5	4
E9Q264_MOUSE	Uncharacterized protein GN=Myh15	0.10	0.44	2.1	4
SODC_MOUSE	Superoxide dismutase [Cu-Zn] GN=Sod1	1.4E-05	2.8E-04	4.7	3
E9QPE7_MOUSE	Uncharacterized protein GN=Myh11	0.08	0.82	3.6	3

Q3UH59_MOUSE	Myosin, heavy polypeptide 10, non-muscle GN=Myh10	0.06	0.37	3.5	3
ANR53_MOUSE	Ankyrin repeat domain-containing protein 53	4.3E-06	0.01	3.2	3
A2A6Q8_MOUSE	Myosin, light polypeptide 4 (Fragment) GN=Myl4	4.2E-12	0.17	3.1	3
Q543D7_MOUSE	Four and a half LIM domains 2, isoform CRA_a GN=Fhl2	4.6E-03	0.04	2.8	3
Q6GT24_MOUSE	Peroxiredoxin 6 GN=Prdx6	5.7E-10	4.8E-03	2.5	3
K6PL_MOUSE	6-phosphofructokinase, liver type GN=Pfkl	1.9E-06	0.01	2.4	3
E9Q607_MOUSE	Uncharacterized protein GN=Actg1	3.3E-04	0.23	2.3	3
H4_MOUSE	Histone H4 GN=Hist1h4a	4.2E-07	4.5E-03	2.2	3
B2RTK3_MOUSE	Histone H2B GN=Hist1h2bm	8.2E-06	0.04	2.2	3
PLIN4_MOUSE	Perilipin-4 GN=Plin4	2.0E-08	8.7E-05	2.0	3
K6PF_MOUSE	6-phosphofructokinase, muscle type GN=Pfkm	4.9E-04	1.6E-03	9.5	2
B2RXX9_MOUSE	Myosin, heavy polypeptide 7, cardiac muscle, beta GN=Myh7	1.4E-15	2.6E-03	8.2	2
E9QLL7_MOUSE	Uncharacterized protein GN=Itga7	3.8E-09	7.9E-05	7.9	2
Q546G4_MOUSE	Albumin 1 GN=Alb	2.5E-06	1.1E-04	7.7	2
RT36_MOUSE	28S ribosomal protein S36, mitochondrial GN=Mrps36	1.7E-08	1.0E-04	7.1	2
F8WIG3_MOUSE	Uncharacterized protein GN=Ninl	3.3E-05	6.0E-03	6.6	2
B2RRX1_MOUSE	Actin, beta GN=Actb	1.0E-05	4.1E-03	6.6	2
LDHC_MOUSE	L-lactate dehydrogenase C chain GN=Ldhc	5.8E-12	1.1E-04	4.6	2
NDUA7_MOUSE	NADH dehydrogenase [ubiquinone] 1 alpha subcomplex subunit 7 GN=Ndufa7	1.7E-24	3.4E-05	4.5	2
E9QAS7_MOUSE	Uncharacterized protein GN=Inpp5a	5.6E-03	0.24	4.2	2
A1AT5_MOUSE	Alpha-1-antitrypsin 1-5 GN=Serpina1e	0.27	0.78	3.5	2
IGH1M_MOUSE	Ig gamma-1 chain C region, membrane-bound form GN=Ighg1	5.4E-18	0.04	3.4	2
D3Z0Z9_MOUSE	Glyceraldehyde-3-phosphate dehydrogenase GN=Gm5069	2.4E-30	4.9E-04	3.3	2
QCR7_MOUSE	Cytochrome b-c1 complex subunit 7 GN=Uqcrb	8.5E-15	8.5E-05	3.1	2
A2AIR5_MOUSE	Glutamate receptor ionotropic, NMDA3A GN=Grin3a	2.3E-05	1.9E-03	3.0	2
A2AEW8_MOUSE	GRIP1 associated protein 1 GN=Gripap1 (neuron-specific guanine nucleotide exchange factor for the Ras family of small G proteins (RasGEF) and is associated with the GRIP/AMPA receptor complex in brain)	3.2E-25	6.4E-04	2.9	2
E9Q1N7_MOUSE	Uncharacterized protein	0.01	0.13	2.7	2
COG6_MOUSE	Conserved oligomeric Golgi complex subunit 6 GN=Cog6	3.2E-03	9.2E-03	2.7	2
E9Q452_MOUSE	Uncharacterized protein GN=Tpm1	2.6E-03	0.01	2.2	2
B1AXW5_MOUSE	Peroxiredoxin 1 (Fragment) GN=Prdx1	1.2E-06	0.03	2.2	2

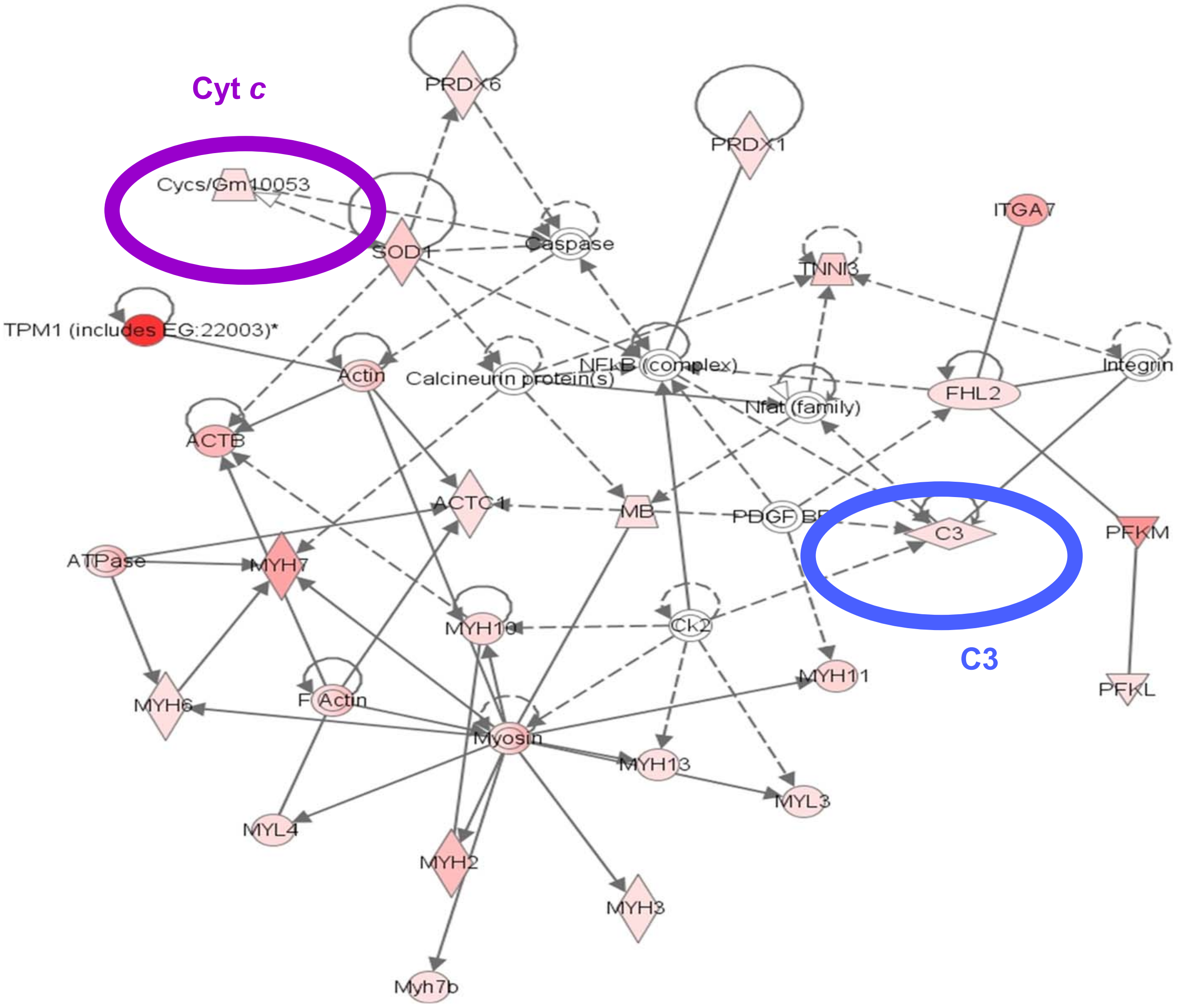

Supplementary Figure S1. Pathway analysis of protein networks associated with the C3. The network was generated by Ingenuity pathway analysis (IPA) software using the list of differentially expressed 57 proteins identified by proteomics. The yellow circle on the left highlights cytochrome *c*, that on the right highlights C3.

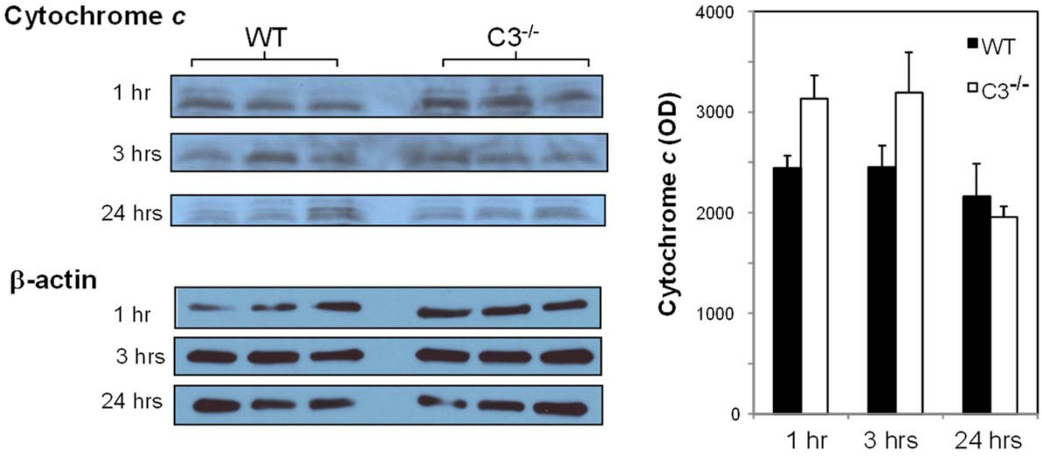

Supplementary Figure S2. Cytochrome *c* levels in C3$^{-/-}$ and WT myocardial cytosols after I/R. Cytosolic factions were isolated and the proteins separated by SDS-PAGE. Western blotting was carried out using an anti-cytochrome *c* Ab and ECL detection. β-actin, detected with an anti-β-actin Ab, acted as a control for cytosol volume added/well. The left panels are blots of cytochrome *c* and β-actin. Each lane represents an individual mouse. The right chart shows the results of densitometric analysis of the cytochrome *c* bands averaged for the 3 mice. $P > 0.05$ for C3$^{-/-}$ and WT mice results.

Proposed experiments in a cell free apoptosis system

Hypotheses	Pre-incubate C3 with Cyt *c*	Pre-incubate C3 with cytosol
C3 binds Cyt *c*	No apoptosis	Apoptosis
C3 binds factors downstream of Cyt *c*	Apoptosis	No apoptosis

Supplementary Figure S3. Schematic diagram depicting the hypothesis and model of the possibilities/results for C3 interaction with factor(s) in the intrinsic apoptotic pathway.

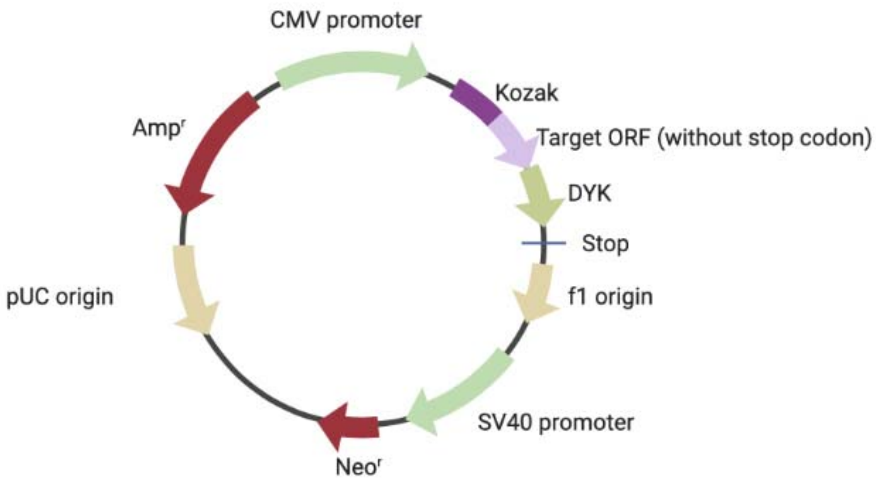

Supplementary Figure S4. pcDNA3.1^{+}/C-(K)DYK vector map. The full-length human C3 expressing clones are constructed using the pcDNA3.1^{+}/C-(K)DYK vector. Target ORF, which is full-length C3 in this study, is cloned into pcDNA3.1^{+}/C-(K)DYK vector without introducing any extra nucleotides before or after target ORF. ORFs cloned in pcDNA3.1^{+}/C-(K)DYK vector were expressed in mammalian cells as a tagged protein with a C-terminal FLAG tag. Proteins expressed from this clone were detected and purified following transgene expression using anti-FLAG antibodies.
